# Left-sided appendicitis revealing a common mesentery: A Case Report

**DOI:** 10.1016/j.radcr.2022.07.009

**Published:** 2022-08-04

**Authors:** Farid Aassouani, Yahya Charifi, Chaymae Hajjar, Nizar El Bouardi, Meryem Haloua, Badreeddine Alami, Moulay Youssef Alaoui Lamrani, Youssef Bouabdallah, Mustapha Maaroufi, Meriem Boubbou

**Affiliations:** a Department of Radiology Mother and Child, CHU Hassan II, FEZ, Sidi Mohammed Ben Abdellah University, Fes, Morocco; b Departement of Infantile General Surgery, CHU Hassan II, FEZ, Sidi Mohammed Ben Abdellah University, Fes, Morocco

**Keywords:** Left appendicitis, Common mesentery, Situs inversus, Left iliac fossa, CT, computerized tomography, LSA, left-sided appendicitis, SIT, situs inversus totalis, SMA, superior mesenteric artery, SMV, superior mesenteric vein

## Abstract

Intestinal malrotation is a congenital rotational anomaly that results of abnormal rotation of the gut, said to occur in 1 in 6000 live births. Common mesentery predisposes to volvulus of the midgut and internal hernias due to the left position of the cecum and appendix. The association of this anomaly with acute left appendicitis is rarely reported in the literature. Occurrence of acute appendicitis on common mesentery is a source of diagnosis difficulties, which may lead to a surgical management delay. We report a case of a 10-year-old boy, admitted for a left-sided iliac pain whose radiological investigations confirmed a left acute appendicitis associated with complete common mesentery. The child underwent laparoscopic surgery with simple post-operative follow-up.

## Introduction

Intestinal malrotation is a rare congenital abnormality estimated to occur in one of 6000 live births [1]. Historically, intestinal malrotation was mainly considered a pediatric disease, and it is rarely diagnosed after the age of 1 year. However, this perception is changing with increased documented presentations above that age [2,3].

Acute appendicitis in this condition has a high risk of missed and delayed diagnosis; therefore, it poses a significant diagnostic challenge [4]. Majority of cases with left sided appendicitis have an associated midgut malrotation, situs inversus totalis or an abnormal position of a long appendix. Due to the ambiguity of symptoms, diagnosis is often difficult and thus increases morbidity and mortality.

Our case focuses on acute appendicitis of left localization with complete common mesentery, which first line of investigations diagnosis was made by ultrasound, and secondary, by contrast-enhanced abdominal CT scan.

## Case presentation

A 10-year-old boy without pathological medical or surgical history, admitted to the pediatric emergency department for a 3 days’ acute abdominal pain. It was not improved by symptomatic treatment with paracetamol.

Clinical examination revealed a soft abdomen with a localized febrile tenderness on the left iliac fossa and hypogastrium. Hemodynamic and respiratory values were normal, they revealed a 129/75 mmHg of blood pressure, and a 95/min heart rate.

Biologically, the complete blood count cell showed a WCC of 24 × 10^9^/L [reference range: 4.5-11.000 × 10^9^/L], a hemoglobin level of 115 g/L [reference range: 10-15.5 g/dl], and a platelet count of 385 × 10^9^ /L [reference range: 150-400^9^ g/dl]. C-reactive protein (CRP) was elevated to 93 mg/L [reference range < 4]; urea, electrolytes, and creatinine were in the normal values.

Abdominal ultrasound does not show appendix on the right iliac fossa; however, it identified a hyperechoic structure generating a posterior acoustic shadowing within dilated tubular structure in the left iliac fossa. There was no intra-abdominal free fluid and the liver, spleen and both kidneys were normal.

**Fig. 1:**
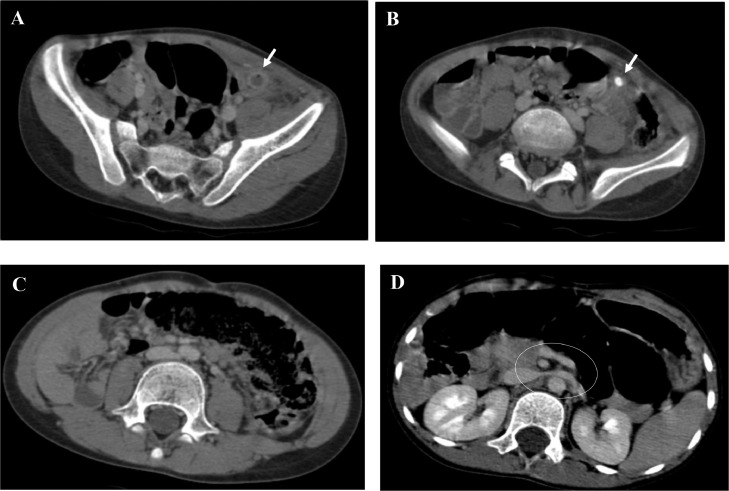
Axial abdominal CT scan (A-B-C) showing: (A) Dilated structure in the left iliac fossa with enhanced mucosa consistent with acute appendicitis (White arrow). (B) Left-sided appendicitis with appendicolith (White arrow). (C) Small bowel on the right and large bowel on the left. (D) Abnormal course of D3 which does not cross the midline (white circle).

Contrast-enhanced abdominal CT scan was performed, revealing a severely inflamed appendix that was significantly dilated to 14 mm in the left iliac fossa with 12 mm appendicolith (Fig. 1). CT scan also demonstrated typical imaging findings of intestinal malrotation including: abnormal position of small intestine located in the right and large bowel in the left, reversal of the normal relationship between the superior mesenteric artery and superior mesenteric vein, and also the absence of a D3 segment that does not cross the median line and continues toward a vertical trajectory.

In this case, we concluded to intestinal malrotation with acute left-sided appendicitis.

**Fig. 2:**
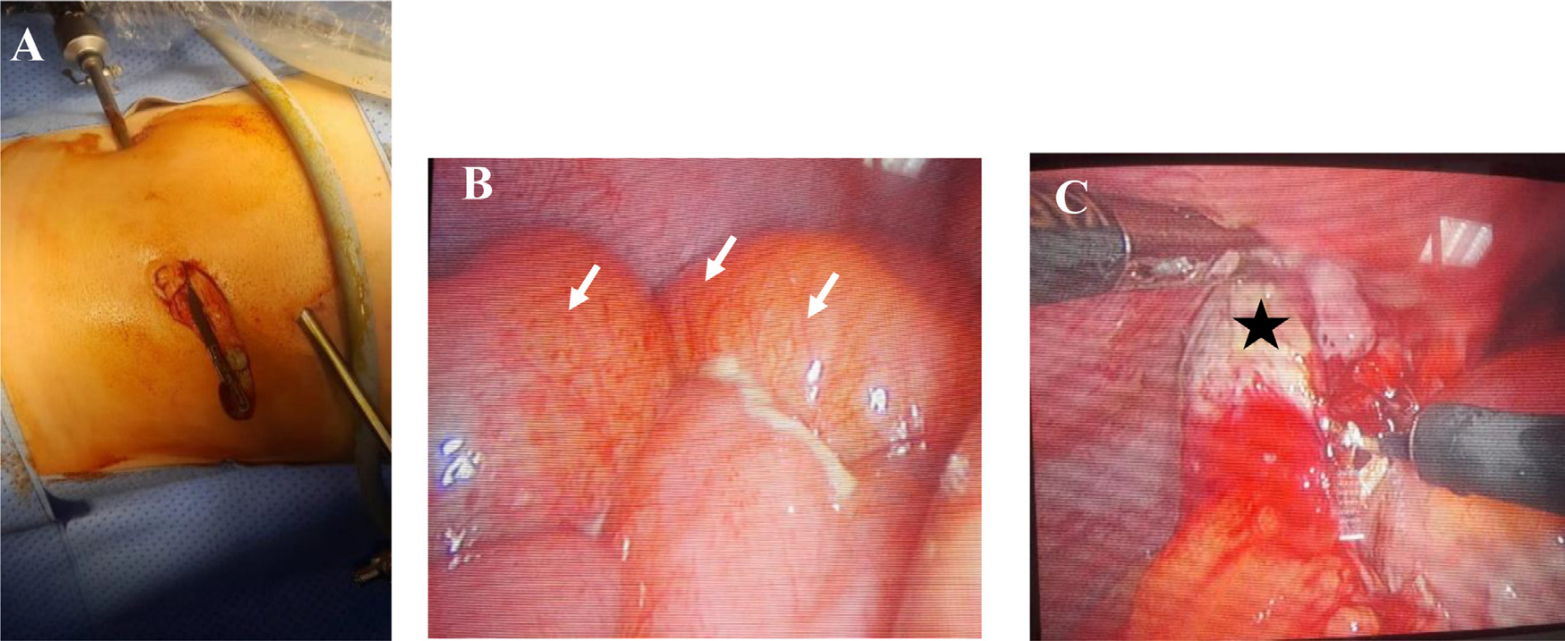
Preoperative findings: introduction of trocars (A), inflamed appendix (B: asterix) and hyperemia of surrounded bowel loops (C: arrows).

An emergency laparoscopic procedure was performed (Fig. 2), and revealed inflamed appendicitis with a stercolith localized in the left iliac fossa which confirmed the initial presumption. There was no associated abscess or free intraperitoneal fluid.

Appendicectomy was done and follow-up was without complications: the child resumed normal transit, the wound was clean and the DELBET blades were removed within 24 hours.

**Fig. 3:**
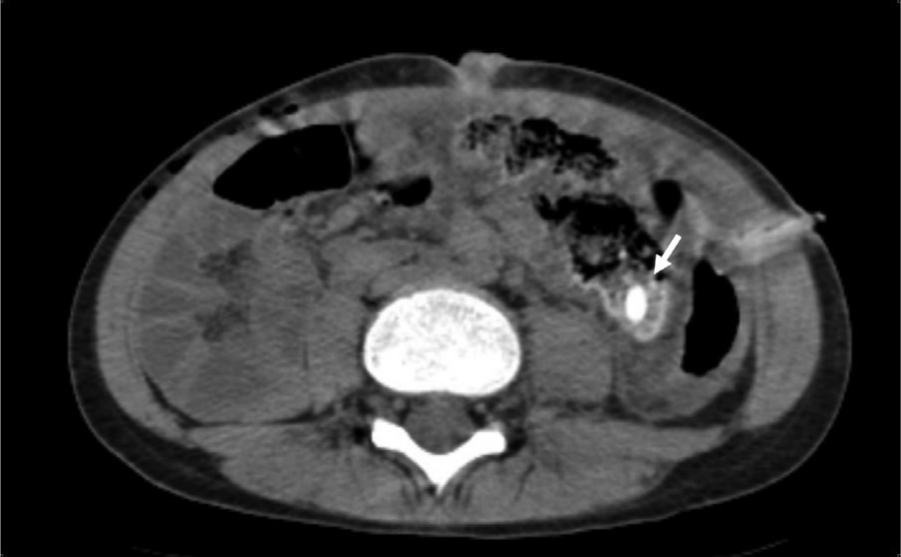
Postoperative abdominal CT scan showing migration of the stercolith in the cecum (white arrow).

Postoperative control CT scan showing the migration of stercolith, currently located at the level of the cecal fundus (Fig. 3).

## Discussion

Several pathological conditions can cause left lower abdominal pain. Digestive causes are the most common especially acute diverticulitis, ulcerative colitis, Crohn's disease and irritable bowel syndrome. Other pathologies may be also considered such as urinary causes, ovarian disease, fallopian inflammation or even a parietal origin [4]. Acute appendicitis is barely considered in the differential diagnosis of left lower abdominal pain.

Acute left iliac pain caused by appendicitis still an ambiguous and challenging diagnosis, and thus, diagnosis is frequently delayed leading to more severe complications.

It is important to know that excessively long right appendicitis that crosses the midline can always mimic a true left appendicitis found in cases of situs inversus or midgut malrotation [5,1].

Midgut malrotation is used to describe a spectrum of congenital positional anomalies of the digestive tract that's results from incomplete rotation of the primitive midgut around the axis of superior mesenteric artery during embryogenesis [6].

Approximately, 50% of cases present in the first week of life while 60% are diagnosed in the first month of life due to the severe complications of midgut volvulus [6]. Other conditions may be diagnosed in childhood or adulthood either due to primitive complications (midgut volvulus / internal hernias) or even incidentally during the investigation of acute or chronic pathologies involving abdominal pain such as in our case [7,8].

Ultrasonographic assessment of common mesentery is proposed as advantageous for children, because aside from its high accuracy, it lacks the radiation effects in comparison with other imaging studies.

Inversion of superior mesenteric vessels is considered to be the key diagnosis of the disease. In a study of 23 patients, Zhou et al. reported sensitivity, specificity, and accuracy of ultrasonography for determining malrotation at 100%, 97.8%, and 98.6%, respectively [9].

CT scan and MRI may confirm SMV and SMA inversion; it can identify a right SMA and a left SMV. This abnormal deviation was originally described by Nichols and Li.

In addition, they may demonstrate the abnormal anatomical arrangements of retro-mesenteric D3 segment of the duodenum which does not cross the spine.

In our case, ultrasound could not confirm the diagnosis, and thus an enhanced abdominal CT scan was carried out, revealing typical features of complete intestinal malrotation.

Situs inversus is the major differential diagnosis of acute appendicitis with common mesentery, it may be complete when there is an inverted position of chest and abdominal organs (situs inversus totalis-SIT) or partial when only one of those cavities is affected.

Some authors such as Collins D reported that the incidence of acute appendicitis associated with SIT after studying 71000 human appendix specimens is 0.016% [5]. In more recent study of 95 cases of LSA published in literature, 69.4% had SIT while 24.2% had midgut malrotation [10].

## Conclusion

LSA is a rare condition that is not usually encountered in the differential diagnosis of left abdominal pain in children. The approach to these patients still a challenging diagnosis, therefore, it can lead to misdiagnosis and medical pitfalls.

Detailed physical examination, laboratory and imaging investigation including U/S and CT scans are the key for a correct diagnosis and safe management and treatment.

## Patient consent

I, the author of the article: “left-sided appendicitis revealing a common mesentery: a case report,” approve that the father of the child, Ali El Khed, gives his consent for information about his child to be published in: Radiology Case Reports.

And thus

He understands that the information will be published without his child name attached, but that full anonymity cannot be guaranteed.

He understands that the text and any pictures or videos published in the article will be freely available on the internet and may be seen by the general public. The pictures, videos and text may also appear on other websites or in print, may be translated into other languages or used for commercial purposes.

He has been offered the opportunity to read the manuscript.

## Ethics approval and consent to participate

Not applicable

## Consent for publication

Written informed consent was obtained from the child parent's, legal guardian for publication of this case report and any accompanying images. A copy of the written consent is available for review by the Editor-in-Chief of this journal.

## Availability of data and materials

The data sets are generated on the data system of the CHU Hassan II of Fes, including the biological data and the interventional report.

## Authors’ contribution

FA is the corresponding author, he participated in the organization and writing of the article and studying the cases with GS.

Professor MB supervised working and validated the figures.

Professor and chief of department of radiology MB and MM red and allowed the article for publication.

All authors read and approved the final manuscript.

## Appendix Supplementary materials

Image, application 1
